# Hereditary Diffuse Gastric Cancer: A 2022 Update

**DOI:** 10.3390/jpm12122032

**Published:** 2022-12-08

**Authors:** Christo Kole, Nikolaos Charalampakis, Stratigoula Sakellariou, George Papaxoinis, Konstantinos G. Apostolou, Nikolaos Machairas, Ioannis S. Papanikolaou, Dimitrios Schizas

**Affiliations:** 1 First Department of Surgery, National and Kapodistrian University of Athens, Laikon General Hospital, 115 27 Athens, Greece; 2 Department of Medical Oncology, Metaxa Cancer Hospital, 185 37 Athens, Greece; 3 First Department of Pathology, National and Kapodistrian University of Athens, 115 27 Athens, Greece; 4 Second Department of Medical Oncology, Agios Savvas Anticancer Hospital, 115 22 Athens, Greece; 5 School of Medicine, European University Cyprus, 2404 Nicosia, Cyprus; 6 Second Department of Propaedeutic Surgery, National and Kapodistrian University of Athens, Laikon General Hospital, 115 27 Athens, Greece; 7 Hepatogastroenterology Unit, Second Department of Internal Medicine and Research Institute, National and Kapodistrian University of Athens, Attikon University Hospital, 124 62 Athens, Greece

**Keywords:** hereditary diffuse gastric cancer, CDH1, CTNNA1, MAP3K6, gastrectomy, clinical guidelines

## Abstract

Gastric cancer is ranked fifth among the most commonly diagnosed cancers, and is the fourth leading cause of cancer-related deaths worldwide. The majority of gastric cancers are sporadic, while only a small percentage, less than 1%, are hereditary. Hereditary diffuse gastric cancer (HDGC) is a rare malignancy, characterized by early-onset, highly-penetrant autosomal dominant inheritance mainly of the germline alterations in the E-cadherin gene (*CDH1*) and β-catenin (*CTNNA1*). In the present study, we provide an overview on the molecular basis of HDGC and outline the essential elements of genetic counseling and surveillance. We further provide a practical summary of current guidelines on clinical management and treatment of individuals at risk and patients with early disease.

## 1. Introduction

Gastric cancer remains an important health issue worldwide, ranking fifth among the most commonly diagnosed malignancies (5.6%) and the fourth leading cause of cancer-related deaths (7.7%); it was responsible for over one million new incidents and an estimated 769,000 deaths in 2020 worldwide [1]. There are two major subtypes of gastric cancer characterized by distinct molecular, morphological and clinical features, the Lauren intestinal/WHO tubular and Lauren diffuse/WHO poorly cohesive subtype [2]. The majority of gastric cancers are sporadic; however, a small percentage (5–10%) arise within inherited or familial cancer syndromes [3]. Among gastric cancers, only 1% to 3% are hereditary [4], including Hereditary Diffuse Gastric Cancer (HDGC), Familial Intestinal Gastric Cancer (FIGC) and Familial Diffuse Gastric Cancer (FDGC) [5,6,7]. Moreover, several syndromes, such as Lynch, Peutz-Jeghers, Li Fraumeni and Familial Adenomatous Polyposis syndromes, are predisposed to the development of gastric cancer [6]. Hereditary Diffuse Gastric Cancer is an autosomal dominant cancer syndrome characterized by an increased risk of diffuse gastric cancer (DGC) and lobular breast cancer (LBC) [8]. It was first described in 1998 in an extended New Zealand Māori family [9], now the prevalence of HDGC is less than 0.1 per 100,000 in the general population and in less than 1% of patients with gastric cancer [10]. HDGC exhibits high penetrance and invasive disease often manifests before age 40; the median age of HDGC diagnosis is 38 years with the overall risk increasing with age [11,12]. The majority of confirmed cases are caused by inactivating germline mutations in the *CDH1* tumor suppressor gene [13]. The lifetime risk of developing gastric cancer in individuals carrying a mutation in *CDH1* gene is estimated to be less than 1% at the age of 20, around 4% by the age of 30, increasing to about 20% for men and 45% for women by the age of 50 [14,15,16,17,18,19]. These mutations in penetrance genes widely vary based on tested populations. In fact, original data were in DGC patients with later reports in laboratory-based populations. It has been shown that HDGC may be responsible for a more aggressive disease phenotype compared to sporadic cancer, with a 5-year survival rate of 4% compared to 13% for sporadic cancer [20,21]. Three distinct tumor cell populations are proposed in HDGC, i.e., well-differentiated large cells, well-differentiated small cells and poorly differentiated small cells; the latter group, with aberrant p16 expression, may represent a more aggressive phenotype [22]. Advanced HDGC is composed by poorly differentiated small cells invading the whole thickness of the gastric wall [22]. In this review, we discuss the available literature on the molecular basis of HDGC and outline the essential elements of genetic counseling and surveillance. Moreover, we refer to recent guidelines on clinical management and treatment recommendations.

## 2. Molecular Basis of HDGC

HDGC, although rare, is an aggressive cancer inherited in an autosomal dominant fashion [23]. The genetic basis was identified in 1998 by Guilford et al., who reported mutations in the E-cadherin gene (*CDH1*) [9]. The human *CDH1* gene is a 2.6 kb tumor suppressor gene located in chromosome 16q22.1 [24,25]. The *CDH1* gene encodes a type I calcium transmembrane glycoprotein expressed on epithelial tissues, responsible for calcium-dependent cell adhesion basolateral membrane in adherent junctions, maintenance of cell differentiation and cell polarity, both during development and in adult life [26,27,28]. The CDH1 120-kDa mature glycoprotein is composed by three domains, an extra-cellular domain containing binding sites for Ca^2+^ ions, a transmembrane domain and a highly conserved cytoplasmic domain containing binding sites for β-catenin (CTNNA1) [26,29]. E-cadherin is an important determinant of tumor progression, serving as a suppressor of invasion and metastasis (Table 1) [30]. The binding of β-catenin prevents its translocation to the nucleus, thus leading to the activation of the Wnt signaling pathway, epithelial-mesenchymal transition process and malignant transformation [31,32,33,34].

Mutated sites in the *CDH1* gene are scattered. There have been over 155 different germline CDH1 mutations identified so far [13], including insertions, deletions and point mutations [12,37]. Moreover, 38% of germline mutations are frameshift mutations, 23% are splice site, 17% are missense and 17% are nonsense mutations affecting the entire coding sequence, resulting in truncated inactive proteins [46]. Almost 90% of the known mutations have been predicted to lead to premature protein truncation or a lack of mRNA expression [47]. Epigenetic factors, such as DNA methylation, have been shown to influence gene expression and serve as the second genetic hit leading to gene inactivation, therefore promoting tumorigenesis [48,49,50]. Oliveira et al. reported that CDH1 epigenetic and genetic alterations were detected in 80% of HDGC families analyzed. Out of 28 HDGC lesions, *CDH1* promoter hypermethylation was found in nine (32.1%) cases [50]. Association between promoter hypermethylation of *CDH1* and gastric cancer was also confirmed in a meta-analysis by Jing et al. [51]. In this direction, demethylating drugs may emerge as therapeutic options in early *CDH1*-driven HDGC stages without evidence of metastatic disease [50]. A clinical trial (NCT04253106), currently in the recruiting stage, aims to detect methylation profiles in asymptomatic carriers who refuse gastrectomy and in controls using liquid biopsies of both blood and gastric fluid [52]. Moreover, post-translational machinery was reported by Figueiredo et al. as a new molecular basis for the loss of E-cadherin [53]. Disruption of the highly conserved hydrophobic core of the signal peptide of E-cadherin impairs the interaction of E-cadherin with cellular components crucial for E-cadherin translation and translocation into the endoplasmic reticulum (ER), decreasing protein synthesis, ER import and membrane activity [53]. Pathogenic E-cadherin missense mutant cells fail to form correct cell-cell adhesions and become more invasive in comparison with cells expressing the wild-type (WT) protein [54,55]. Mutations in *CDH1* gene have been associated with the development of signet ring cell carcinoma in situ (SRCC-pTis), characterized by tumor cells located between normal foveolar epithelial cells and the basement membrane. SRCC-pTis is considered the precursor lesion of HDGC [56,57]. Penetrance of HDGC in CDH1 mutated carriers is approximately 40% to 67% for men and 63% to 83% for women [12,58]. The cumulative incidence of HDGC by the age of 80 years is 70% (95% CI, 59–80%) for males and 56% (95% CI, 44–69%) for females [13], while the combined risk is estimated at 80% [11,15].

Another gene implicated in HDGC is *CTNNA1* (Table 1). The human *CTNNA1* gene is located in chromosome 5q31.2, encoding a member of the catenin family that plays an important role in cell adhesion to the actin filaments inside the cell [59]. The A-catenin family consists of three members, aE-catenin, aN-catenin and aT-catenin expressed in epithelia, neurons and testis—heart, respectively, playing an important role in cell adhesion by connecting to cadherins [59,60,61]. A lack of function of CTNNA1 leads to the destabilization of adherent junctions, making cells less sensitive to cell contact-mediated inhibition, thus facilitating cell migration and invasion [62,63,64]. This correlates with poor prognosis. Pathogenic variants of *CTNNA1* have been described in 14 families [38,39]. In the United States, all *CTNNA1* variants commercially used in the laboratory are classified as variants of uncertain significance, including *CTNNA1 loss-of-function* (LOF) variants, thus justifying the low numbers [39]. Thus far, only one frameshift mutation has been detected leading to a germline truncating allele of α-E-catenin in family members with invasive HDGC, and four in patients with intramucosal signet ring cells detected as part of endoscopic surveillance [13,37]. Majewski et al. reported a 2 bp deletion in exon 2, which results in a frameshift mutation after Arg27 and truncated *CTNNA1* gene [37]. In all cases, the onset of HDGC occurred after the age of 50 [13,37]. Moreover, rare germline missense mutations in other genes (Table 1) [13] of high and moderate penetrance have been previously reported as pathogenic. Included genes are: insulin receptor (INSR) [40], which has been shown to affect tumor cell invasion by modulating E-cadherin glycosylation [65,66]; F-box protein 24 (FBXO24) [40], which may regulate cell proliferation by mediating ubiquitination and degradation of PRMT6 [67]; DOT1-like histone H3K79 methyltransferase (DOT1L) [40], which is required for proper DNA damage response [68]; mitogen-activated protein 3-kinase 6 (MAP3K6) [41], which acts as a tumor suppressor gene [41]; the pathogenic missense variant (F354L) in the highly penetrant Peutz-Jeghers syndrome susceptibility gene, serine/threonine kinase 11 (STK11) (also called LKB1) [43,44]; and the partner and localizer of BRCA2 (PALB2) [43,44,45], which have also been implicated in HDGC occurrence. However, Weren et al. concluded that *MAP3K6* should no longer be considered a gastric cancer predisposition gene since, according to their data [42], two missense variants, which have previously been associated with familial gastric cancer, are also frequently observed in the control data set and no significant differences between the two datasets were found.

## 3. Genetic Testing and Counseling

The International Gastric Linkage Consortium (IGCLC) set criteria to provide critical information for medical care providers and for those patients and families at risk of HDGC syndrome. The 2020 updated guidelines [8] recommend genetic testing in individuals who have a confirmed cancer diagnosis and meet one of the following family criteria (Table 2): (1) two or more gastric cancer cases, regardless of age, with at least one histologically confirmed diffuse gastric cancer (DGC); (2) one or more cases of DGC at any age, and one or more cases of LBC at an age less than 70 years, in different family members; and (3) two or more cases of LBC in family members less than 50 years of age. Individual criteria include: confirmed DGC at an age less than 50 years; DGC at any age in individuals of Māori ethnicity; DGC at any age in individuals with a personal or family history (first-degree relative) of cleft lip/cleft palate; history of DGC and LBC both diagnosed at an age earlier than 70 years; bilateral LBC diagnosed at an age earlier than 70 years; and in situ gastric signet ring cells or pagetoid spread of signet ring cells in individuals less than 50 years [8,69].

Hereditary cancer syndromes presuppose an elevated risk of cancer in family members [3]; therefore, before testing, genetic counseling for HDGC should take place by a team of experts including a gastroenterologist, a geneticist, a surgeon and an oncologist. All possible outcomes of testing and the associated management options should be discussed. It is suggested that individuals who fulfill the criteria for HDGC should be offered genetic testing from the legal age of consent (18 years) [70]; testing should be performed first for *CDH1* mutations, and, if there is no variant identified, then they should subsequently be considered for *CTNNA1* analysis [71]. However, age of testing should also consider the earliest age of cancer onset in HDGC families from the local population. For example, in New Zealand, *CDH1* mutation carriers have developed gastric cancer in their mid-teens; as a consequence, genetic testing begins at 16 years of age, and occasionally 1–2 years before, on a case-by-case basis [15]. Using the criteria for HDGC, the detection rate of *CDH1* pathogenic or likely pathogenic variants is detected in 48% of families with multiple cases of gastric cancer [72]. Genetic evaluation should include a three-generation family pedigree, histopathological confirmation of DGC diagnoses or precursor lesions, and a discussion of lifetime risks of DGC and LBC and current *CDH1* mutation detection rates [11,12,50]. Analysis for *CDH1* and *CTNNA1* germline variants must include sequencing of the entire open reading frame (ORF), including intron-exon boundaries [8]. Germline *CDH1* point or small frameshift mutations can be identified in 30–50% of HDGC families, while *CDH1* large deletions account for less than 5% of pathogenic variants [47,73]. Available variant information can be obtained from the LOVD team [69] in the locus-specific database [74]. Genetic testing should include copy number analysis of exons to detect deletions or duplications of the *CDH1* gene [8]. Results interpretation should be performed using the ACMG/AMP guidelines [75,76]. As pathogenic germline *CDH1* variants have high prevalence in the New Zealand Māori population, all Māori with confirmed DGC are recommended to undergo *CDH1* genetic testing [77]. Moreover, individuals with a personal or family history of cleft lip/palate and DGC or with HDGC precursor lesions are recommended to undergo genetic testing [69,78]. Initially, genetic testing should be performed in an affected patient in order to identify the genetic alteration. Thereafter, first-degree family members may be analyzed for that specific mutation [57,79]. In case no living affected family member exists, formalin-fixed paraffin-embedded specimens from the deceased relative may be performed [79]. However, this is not the common method for most laboratories. Nevertheless, it is worth mentioning that no genetic testing should be performed in patients with no family history of cancer and no previous genetic counseling [80].

Concerning molecular genetic analysis approaches, single-gene testing is recommended followed by gene-targeted deletion/duplication analysis if no pathogenic variant is found [81]. A multigene panel testing (MGPT) that includes *CDH1* and other genes of interest may also be considered using next generation sequencing techniques searching for mutations [39,82,83,84]. The MGPT is a type of genetic testing that looks for mutations in several genes at once, and has become a critical component of cancer risk assessment in clinical practice [85]. Other methods used may include quantitative PCR, long-range PCR, multiplex ligation-dependent probe amplification (MLPA), and a gene-targeted microarray designed to detect single exon deletions or duplications [81]. Using the American College of Medical Genetics and Genomics and the Association for Molecular Pathology updated guidelines, germline sequence variants found in genetic analysis are classified as: (a) pathogenic; (b) likely pathogenic; (c) variant of uncertain significance; (d) likely benign; and (e) benign [75,86,87].

HDGC is associated with LBC which may be the presenting pathology in a patient [88]. The results presented by S. Masciari et al. were not isolated cases, but rather cases of familial LBC. LBC is characterized by small, dyscohesive, diffusely infiltrating epithelial cells which do not form a well-defined mass and have a high predisposition to metastasize to the gastrointestinal and peritoneal surface, as well as to the female reproductive tract [79]. LBC is frequently multicentric and bilateral compared to ductal breast cancer, and bilateral prophylactic mastectomy is usually suggested in asymptomatic individuals with a strong family history of LBC and a *CDH1* pathogenic variant [69]. In contrast to ductal breast carcinomas, approximately 90% of invasive LBCs are estimated to display E-cadherin loss [89]. Interestingly, the estimated cumulative risk for LBC in *CDH1* mutation female carriers is 39% (95%CI, 12–84%) while the combined risk of gastric cancer and breast cancer in women rises to 90% by age 80 years, indicating that family history of multiple LBCs at a young age should encourage genetic screening for *CDH1* mutations [11]. The American College of Gastroenterology also recommends breast cancer screening for women with HDGC through annual mammography and semiannual breast MRI, with breast examinations starting at the age of 35 years [8,90] in concordance with the National Comprehensive Cancer Network (NCCN) guidelines [91]. Bilateral LBC under the age of 50 years or in at least two close relatives under the age of 50 years [92,93], as well as individuals with a personal or family history of cleft lip/cleft palate and DGC [78,94,95], may justify testing for *CDH1* mutations.

## 4. Clinical Surveillance and Management

Management of patients with symptomatic invasive HDGC is not different to that of sporadic cases [96,97,98,99]. Prognosis in patients with widely invasive DGC is very poor, with less than 10% of them being potentially curable [100]. Furthermore, the 5-year survival rate is still less than 30%, even for the potentially curable DGC [101], which does not differ much from sporadic gastric cancer [102,103]. On the other hand, despite the fact that prophylactic gastrectomy has a post-operative mortality rate of approximately 1% and is a life-changing procedure reducing quality of life (risks of infections, dumping syndrome and weight loss) [58,104], it has a curative result. When gastric cancer is detected early and resected by total gastrectomy, the 5-year survival rate is 90% [81,105]. For patients with metastatic cancer, a systemic treatment is recommended after testing for the expression of human epidermal growth factor receptor-2 (HER-2) in order to check whether trastuzumab should be added to well-established chemotherapeutic regimens [106]. The most commonly used first-line chemotherapy regimens for metastatic disease combine a platinum and a fluoropyrimidine compound, such as FOLFOX, CAPOX, cisplatin/5-fluorouracil (5-FU) or cisplatin/capecitabine [98,107]. These combinations have demonstrated prolongation of survival by approximately 7 months compared to palliative care alone [97]. Recent advances in immunotherapy have revolutionized the treatment of oncological patients [108,109,110,111]. Immunotherapy utilizes monoclonal antibodies to target tumor-produced immune checkpoint receptors, such as Programed cell Death protein-1 (PD-1), Programed cell Death protein Ligand-1 (PD-L1) and Cytotoxic T-Lymphocyte–associated Antigen 4 (CTLA-4) that prevent T cells from recognizing and eliminating cancer cells [112,113]. Recently, concerning the initial treatment of patients with advanced or metastatic gastric cancer, the addition of immunotherapy, namely of PD-1 inhibitor nivolumab to chemotherapy, achieved a survival benefit after more than a decade [114]. Moreover, the anti-PD1 antibody pembrolizumab has been approved for the treatment of patients with advanced, recurrent or metastatic gastric cancer with either microsatellite instability (MSI-H) or DNA mismatch repair deficiency (dMMR) [115]. Importantly, differential high-throughput drug screening of c.1380delA CDH1 SB.mhdgc-1 from HDGC patients versus sporadic gastric cancer cells identified increased sensitivity to EGFR inhibitors, including mTOR (Mammalian Target Of Rapamycin), MEK (Mitogen-Activated Protein Kinase), c-Src kinase, FAK (Focal Adhesion Kinase), PKC (Protein Kinase C) and TOPO2 (Topoisomerase II) inhibitors [116], suggesting that anti-mTOR, anti-PI3K and anti-EGFR therapies should be clinically trialed in HDGC patients. Moreover, Beetham et al. reported the identification of two novel compounds with significant synthetic lethal activity that may provide a new strategy for the prevention and treatment of both sporadic and hereditary LBC and DGC [117].

Moreover, there have been several studies on cell-therapy applications in solid tumors. Chimeric antigen receptor (CAR)-T cell immunotherapy using genetic engineering to harness the anti-tumor activity of T-lymphocytes. T cells from the patients’ bloodstream were modified using viral vectors to introduce the CAR to recognize a particular tumor antigen and then were infused back into the patient [118]. Promising results have been shown in preclinical models where engineering of CAR-T cells with integrin αEβ7 results in augmented therapeutic efficacy against E-cadherin positive tumors [119]. In addition, CD24 is an attractive prognostic factor for HDGC [120], and therefore, a potential target for CAR-T cells [121]. Nevertheless, a number of factors result in a reduction in the effectiveness of CAR-T cell therapy in solid tumors, including immunosuppressive tumor microenvironment (TME). TME significantly weakens T-cell function by overexpressing inhibitory receptors and immunosuppressive cells, such as regulatory T-cells (Tregs); and tumor-associated neutrophils (TAN); myeloid-derived suppressor cells (MDSC); and tumor-associated macrophages (TAM), which facilitate tumor immune escape [122]. On the other hand, IL-12 has been proved efficacious in reversing the TME to Th1 anti-tumor phenotype [123]. In addition, E-cadherin also plays a crucial role in negatively regulating Wnt signaling [124] which in turn is correlated with the inhibition of migration and invasion of GC cells [125]. Moreover, down-regulation of Wnt/β-catenin is correlated with an increased sensitivity of GC cells to PD-1 antibody [126] providing new options also for HDGC therapeutic interventions in the future.

## 5. Conclusions

Despite the fact that HDGC is a rare cancer syndrome, it is highly penetrant and difficult to diagnose and manage. HDGC requires the successful collaboration of a team of experts, including a geneticist, a gastroenterologist, a surgeon and an oncologist for the best decision making at each stage of disease, in order to achieve optimal long-term results. The IGCLC 2010 criteria have evolved to help clinicians make the most effective decisions. Genetic counseling should be performed before testing, while it is crucial to identify a mutation and to further determine whether unaffected relatives are at risk for cancer. There has also been progress in endoscopic techniques for the surveillance of patients who wish to postpone surgery or for those whose risk is not well defined. Nevertheless, endoscopy does not appear to offer a reliable reduction of risk in individuals with a proven pathogenic germline *CDH1* mutation. Therefore, prophylactic total gastrectomy remains the cornerstone of HDGC management. However, the impact of gastrectomy should not be underestimated, since it may have severe consequences on a metabolic and nutritional level and, therefore, patients’ quality of life. Importantly, significant progress has been made in recent years in the identification of *CDH1* mutations. Future efforts should be directed towards the development of improved genetic screening modalities and the identification of new genes, especially in *CDH1*-negative families, aiming at the early identification of asymptomatic carriers.

## Figures and Tables

**Table 1:** Molecular basis of HDGC.

Gene	Corresponding Protein	Functions	Mutation Type	Lifetime Risk of HDGC/Comments	Ref.
*CDH1*	E-cadherin	cell adhesion; tumor suppressor; cell maturation	Frameshift, Splice site, Missense, Nonsense, Large rearrangements	Almost 40% of the cases	[9,13,35,36]
*CTNNA1*	alpha-E-catenin	cell adhesion; signal transduction	Nonsense	Almost 40% of the cases	[13,37,38,39]
*INSR*	Insulin receptor	affect tumor cell invasion; E-cadherin glycosylation	Missense		[40]
*FBXO24*	F-box protein 24	cell proliferation; ubiquitination and degradation of PRMT6	Missense		[40]
*DOT1L*	DOT1-like histone H3K79 methyltransferase	effect on DNA repair	Missense		[40]
*MAP3K6*	Mitogen-activated protein kinase kinase kinase 6	tumor suppressor gene	Nonsense, Missense	Controversial results	[41,42]
*STK11*	serine/threonine kinase 11	tumor suppressor	Missense		[43,44]
PALB2	Partner and localizer of BRCA2	regulating homologous DNA recombination	Nonsense		[43,44,45]

**Table 2:** Criteria for genetic evaluation for HDGC in affected families according to ACG 2020 updated clinical guidelines [8,69].

Individuals with a Confirmed Cancer Diagnosis Plus One of:	
	I. ≥2 cases of DGC cases regardless of age, with at least one confirmed histologically for DGC
	II. ≥1 cases of DGC at any age, and one or more cases of LBC at age less than 70 years, in different family members
	III. ≥2 cases of LBC in family members less than 50 years
Confirmed DGC at age less than 50 years of age	
DGC at any age in individuals of Māori ethnicity	
DGC at any age in individuals with a personal or family history (first-degree relative) of cleft lip/cleft palate	
History of DGC and LBC both diagnosed at age earlier than 70 years	
Bilateral LBC diagnosed at age earlier than 70 years	
In situ gastric signet ring cells or pagetoid spread of signet ring cells in individuals less than 50 years of age	

HDGC: Hereditary diffuse gastric cancer; DGC: Diffuse gastric cancer; LBC: Lobular breast cancer.

## Data Availability

Not applicable.

## References

[1] Sung H., Ferlay J., Siegel R.L., Laversanne M., Soerjomataram I., Jemal A., Bray F. (2021). Global cancer statistics 2020: Globocan estimates of incidence and mortality worldwide for 36 cancers in 185 countries. CA Cancer J. Clin..

[2] Classification of Tumours Editorial Board (2019). Digestive System Tumours.

[3] American College of Obstetricians and Gynecologists (2019). Hereditary Cancer Syndromes and Risk Assessment: Acog Committee Opinion, Number 793. Obstet. Gynecol..

[4] Van der Kaaij R.T., Koemans W.J., van Putten M., Snaebjornsson P., Luijten J.C., van Dieren J.M., Cats A., Lemmens V.E., Verhoeven R.H.A., van Sandick J.W. (2020). A population-based study on intestinal and diffuse type adenocarcinoma of the oesophagus and stomach in the Netherlands between 1989 and 2015. Eur. J. Cancer.

[5] Stoffel E.M. (2016). Heritable Gastrointestinal Cancer Syndromes. Gastroenterol. Clin. N. Am..

[6] Colvin H., Yamamoto K., Wada N., Mori M. (2015). Hereditary Gastric Cancer Syndromes. Surg. Oncol. Clin. N. Am..

[7] Carneiro F., Oliveira C., Suriano G., Seruca R. (2007). Molecular pathology of familial gastric cancer, with an emphasis on hereditary diffuse gastric cancer. J. Clin. Pathol..

[8] Blair V.R., McLeod M., Carneiro F., Coit D.G., D’Addario J.L., van Dieren J.M., Harris K.L., Hoogerbrugge N., Oliveira C., van der Post R.S. (2020). Hereditary diffuse gastric cancer: Updated clinical practice guidelines. Lancet Oncol..

[9] Guilford P., Hopkins J., Harraway J., McLeod M., McLeod N., Harawira P., Taite H., Scoular R., Miller A., Reeve A.E. (1998). E-cadherin germline mutations in familial gastric cancer. Nature.

[10] Oliveira C., Seruca R., Hoogerbrugge N., Ligtenberg M., Carneiro F. (2013). Clinical utility gene card for: Hereditary diffuse gastric cancer (HDGC). Eur. J. Hum. Genet..

[11] Pharoah P.D., Guilford P., Caldas C., International Gastric Cancer Linkage Consortium (2001). Incidence of gastric cancer and breast cancer in CDH1 (E-cadherin) mutation carriers from hereditary diffuse gastric cancer families. Gastroenterology.

[12] Kaurah P., MacMillan A., Boyd N., Senz J., De Luca A., Chun N., Suriano G., Zaor S., Van Manen L., Gilpin C. (2007). Founder and Recurrent CDH1 Mutations in Families With Hereditary Diffuse Gastric Cancer. JAMA.

[13] Hansford S., Kaurah P., Li-Chang H., Woo M., Senz J., Pinheiro H., Schrader K.A., Schaeffer D.F., Shumansky K., Zogopoulos G. (2015). Hereditary Diffuse Gastric Cancer Syndrome: CDH1 Mutations and Beyond. JAMA Oncol..

[14] Pandalai P.K., Lauwers G.Y., Chung D.C., Patel D., Yoon S.S. (2011). Prophylactic total gastrectomy for individuals with germline CDH1 mutation. Surgery.

[15] Fitzgerald R.C., Hardwick R., Huntsman D., Carneiro F., Guilford P., Blair V., Chung D.C., Norton J., Ragunath K., Van Krieken J.H. (2010). Hereditary diffuse gastric cancer: Updated consensus guidelines for clinical management and directions for future research. J. Med. Genet..

[16] Oliveira C., Seruca R., Carneiro F. (2006). Genetics, Pathology, and Clinics of Familial Gastric Cancer. Int. J. Surg. Pathol..

[17] Yamamoto E., Suzuki H., Takamaru H., Yamamoto H., Toyota M., Shinomura Y. (2011). Role of DNA Methylation in the Development of Diffuse-Type Gastric Cancer. Digestion.

[18] Suriano G., Yew S., Ferreira P., Senz J., Kaurah P., Ford J.M., Longacre T.A., Norton J.A., Chun N., Young S. (2005). Characterization of a Recurrent Germ Line Mutation of the *E-Cadherin* Gene: Implications for Genetic Testing and Clinical Management. Clin. Cancer Res..

[19] Blair V., Martin I., Shaw D., Winship I., Kerr D., Arnold J., Harawira P., McLeod M., Parry S., Charlton A. (2006). Hereditary Diffuse Gastric Cancer: Diagnosis and Management. Clin. Gastroenterol. Hepatol..

[20] van der Post R.S., Vogelaar I.P., Manders P., van der Kolk L.E., Cats A., van Hest L.P., Sijmons R., Aalfs C.M., Ausems M.G., Gomez García E.B. (2015). Accuracy of Hereditary Diffuse Gastric Cancer Testing Criteria and Outcomes in Patients With a Germline Mutation in CDH1. Gastroenterology.

[21] Han M.A., Oh M.G., Choi I.J., Park S.R., Ryu K.W., Nam B.-H., Cho S.-J., Kim C.G., Lee J.H., Kim Y.-W. (2012). Association of Family History With Cancer Recurrence and Survival in Patients With Gastric Cancer. J. Clin. Oncol..

[22] Lee H.E., Smyrk T.C., Zhang L. (2018). Histologic and immunohistochemical differences between hereditary and sporadic diffuse gastric carcinoma. Hum. Pathol..

[23] Stone J., Bevan S., Cunningham D., Hill A., Rahman N., Peto J., Marossy A., Houlston R.S. (1999). Low frequency of germline E-cadherin mutations in familial and nonfamilial gastric cancer. Br. J. Cancer.

[24] Mansouri A., Spurr N., Goodfellow P.N., Kemler R. (1988). Characterization and chromosomal localization of the gene encoding the human cell adhesion molecule uvomorulin. Differentiation.

[25] Berx G., Staes K., van Hengel J., Molemans F., Bussemakers M.J., van Bokhoven A., van Roy F. (1995). Cloning and characterization of the human invasion suppressor gene E-cadherin (CDH1). Genomics.

[26] Van Roy F. (2014). Beyond E-cadherin: Roles of other cadherin superfamily members in cancer. Nat. Rev. Cancer.

[27] Pećina-Šlaus N. (2003). Tumor suppressor gene E-cadherin and its role in normal and malignant cells. Cancer Cell Int..

[28] Priest A.V., Shafraz O., Sivasankar S. (2017). Biophysical basis of cadherin mediated cell-cell adhesion. Exp. Cell Res..

[29] Gall T.M.H., Frampton A.E. (2013). Gene of the month: E-cadherin (*CDH1*). J. Clin. Pathol..

[30] Jeanes A., Gottardi C.J., Yap A.S. (2008). Cadherins and cancer: How does cadherin dysfunction promote tumor progression?. Oncogene.

[31] Polakis P. (2012). Wnt Signaling in Cancer. Cold Spring Harb. Perspect. Biol..

[32] Flanagan D.J., Austin C.R., Vincan E., Phesse T.J. (2018). Wnt Signalling in Gastrointestinal Epithelial Stem Cells. Genes.

[33] Valenta T., Hausmann G., Basler K. (2012). The many faces and functions of β-catenin. EMBO J..

[34] Orsulic S., Huber O., Aberle H., Arnold S., Kemler R. (1999). E-cadherin binding prevents beta-catenin nuclear localization and beta-catenin/LEF-1-mediated transactivation. J. Cell Sci..

[35] Gayther S.A., Gorringe K., Ramus S., Huntsman D., Roviello F., Grehan N., Machado J.C., Pinto E., Seruca R., Halling K. (1998). Identification of germ-line E-cadherin mutations in gastric cancer families of European origin. Cancer Res..

[36] Berx G., Becker K.F., Hofler H., van Roy F. (1998). Mutations of the human E-cadherin (*CDH1*) gene. Hum. Mutat..

[37] Majewski I.J., Kluijt I., Cats A., Scerri T.S., de Jong D., Kluin R.J., Hansford S., Hogervorst F.B., Bosma A.J., Hofland I. (2013). An α-E-catenin (*CTNNA1*) mutation in hereditary diffuse gastric cancer. J. Pathol..

[38] Benusiglio P.R., Colas C., Guillerm E., Canard A., Delhomelle H., Warcoin M., Bellanger J., Eyries M., Zizi M., Netter J. (2019). Clinical implications of CTNNA1 germline mutations in asymptomatic carriers. Gastric Cancer.

[39] Clark D.F., Michalski S.T., Tondon R., Nehoray B., Ebrahimzadeh J., Hughes S.K., Soper E.R., Domchek S.M., Rustgi A.K., Pineda-Alvarez D. (2020). Loss-of-function variants in CTNNA1 detected on multigene panel testing in individuals with gastric or breast cancer. Genet. Med..

[40] Donner I., Kiviluoto T., Ristimäki A., Aaltonen L.A., Vahteristo P. (2015). Exome sequencing reveals three novel candidate predisposition genes for diffuse gastric cancer. Fam. Cancer.

[41] Gaston D., Hansford S., Oliveira C., Nightingale M., Pinheiro H., MacGillivray C., Kaurah P., Rideout A.L., Steele P., Soares G. (2014). Germline Mutations in MAP3K6 Are Associated with Familial Gastric Cancer. PLoS Genet..

[42] A Weren R.D., van der Post R.S., Vogelaar I.P., van Krieken J.H., Spruijt L., Lubinski J., Jakubowska A., Teodorczyk U., Aalfs C.M., van Hest L.P. (2018). Role of germline aberrations affecting *CTNNA1*, *MAP3K6* and *MYD88* in gastric cancer susceptibility. J. Med. Genet..

[43] Fewings E., Larionov A., Redman J., A Goldgraben M., Scarth J., Richardson S., Brewer C., Davidson R., Ellis I., Evans D.G. (2018). Germline pathogenic variants in PALB2 and other cancer-predisposing genes in families with hereditary diffuse gastric cancer without CDH1 mutation: A whole-exome sequencing study. Lancet Gastroenterol. Hepatol..

[44] Sahasrabudhe R., Lott P., Bohorquez M., Toal T., Estrada A.P., Suarez J.J., Brea-Fernández A., Cameselle-Teijeiro J., Pinto C., Ramos I. (2017). Germline Mutations in PALB2, BRCA1, and RAD51C, Which Regulate DNA Recombination Repair, in Patients With Gastric Cancer. Gastroenterology.

[45] Carreño M., Pena-Couso L., Mercadillo F., Perea J., Urioste M. (2020). Investigation on the Role of PALB2 Gene in CDH1-Negative Patients With Hereditary Diffuse Gastric Cancer. Clin. Transl. Gastroenterol..

[46] Oliveira C., Pinheiro H., Figueiredo J., Seruca R., Carneiro F. (2015). Familial gastric cancer: Genetic susceptibility, pathology, and implications for management. Lancet Oncol..

[47] Oliveira C., Senz J., Kaurah P., Pinheiro H., Sanges R., Haegert A., Corso G., Schouten J., Fitzgerald R., Vogelsang H. (2009). Germline CDH1 deletions in hereditary diffuse gastric cancer families. Hum. Mol. Genet..

[48] Grady W.M., Willis J., Guilford P.J., Dunbier A.K., Toro T.T., Lynch H.T., Wiesner G.L., Ferguson K., Eng C., Park J.-G. (2000). Methylation of the CDH1 promoter as the second genetic hit in hereditary diffuse gastric cancer. Nat. Genet..

[49] Barber M., Murrell A., Ito Y., Maia A.-T., Hyland S., Oliveira C., Save V., Carneiro F., Paterson A., Grehan N. (2008). Mechanisms and sequelae of E-cadherin silencing in hereditary diffuse gastric cancer. J. Pathol..

[50] Oliveira C., Sousa S., Pinheiro H., Karam R., Bordeira-Carriço R., Senz J., Kaurah P., Carvalho J., Pereira R., Gusmão L. (2009). Quantification of Epigenetic and Genetic 2nd Hits in CDH1 During Hereditary Diffuse Gastric Cancer Syndrome Progression. Gastroenterology.

[51] Jing H., Dai F., Zhao C., Yang J., Li L., Kota P., Mao L., Xiang K., Zheng C., Yang J. (2014). Association of Genetic Variants in and Promoter Hypermethylation of CDH1 With Gastric Cancer: A meta-analysis. Medicine.

[52] Paris A.P.-H.d. Liquid Biopsies for the Personalized Management of Patients With Hereditary Diffuse Gastric Cancer. https://ClinicalTrials.gov/show/NCT04253106.

[53] Figueiredo J., Melo S., Gamet K., Godwin T., Seixas S., Sanches J.M., Guilford P., Seruca R. (2018). E-cadherin signal sequence disruption: A novel mechanism underlying hereditary cancer. Mol. Cancer.

[54] Figueiredo J., Söderberg O., Simoes-Correia J., Grannas K., Suriano G., Seruca R. (2013). The importance of E-cadherin binding partners to evaluate the pathogenicity of E-cadherin missense mutations associated to HDGC. Eur. J. Hum. Genet..

[55] Simões-Correia J., Figueiredo J., Lopes R., Stricher F., Oliveira C., Serrano L., Seruca R. (2012). E-Cadherin Destabilization Accounts for the Pathogenicity of Missense Mutations in Hereditary Diffuse Gastric Cancer. PLoS ONE.

[56] Tsugeno Y., Nakano K., Nakajima T., Namikawa K., Takamatsu M., Yamamoto N., Fujisaki J., Nunobe S., Kitagawa M., Takeuchi K. (2020). Histopathologic Analysis of Signet-ring Cell Carcinoma In Situ in Patients With Hereditary Diffuse Gastric Cancer. Am. J. Surg. Pathol..

[57] Sugimoto S., Komatsu H., Morohoshi Y., Kanai T. (2015). Recognition of and recent issues in hereditary diffuse gastric cancer. J. Gastroenterol..

[58] Caldas C., Carneiro F., Lynch H.T., Yokota J., Wiesner G.L., Powell S.M., Lewis F.R., Huntsman D.G., Pharoah P.D., Jankowski J. (1999). Familial gastric cancer: Overview and guidelines for management. J. Med. Genet..

[59] Simon-Chazottes D., Ringwald M., Kemler R. (1995). The genes coding for alpha and beta catenin (Catna1 and Catnb) and Plakoglobin (Jup) map to mouse Chromosomes 18, 9, and 11, respectively. Mamm. Genome.

[60] Pokutta S., Weis W.I. (2000). Structure of the Dimerization and β-Catenin- Binding Region of α-Catenin. Mol. Cell.

[61] Pokutta S., Choi H.-J., Ahlsen G., Hansen S.D., Weis W.I. (2014). Structural and Thermodynamic Characterization of Cadherin·β-Catenin·α-Catenin Complex Formation. J. Biol. Chem..

[62] Benjamin J.M., Nelson W.J. (2008). Bench to bedside and back again: Molecular mechanisms of α-catenin function and roles in tumorigenesis. Semin. Cancer Biol..

[63] Chi Q., Xu H., Song D., Wang Z., Wang Z., Ma G. (2020). α-E-Catenin (CTNNA1) Inhibits Cell Proliferation, Invasion and EMT of Bladder Cancer. Cancer Manag. Res..

[64] De Groot J.S., Ratze M.A., Van Amersfoort M., Eisemann T., Vlug E.J., Niklaas M.T., Chin S.-F., Caldas C., van Diest P.J., Jonkers J. (2018). αE-catenin is a candidate tumor suppressor for the development of E-cadherin-expressing lobular-type breast cancer. J. Pathol..

[65] De-Freitas-Junior J.C.M., Carvalho S., Dias A.M., Oliveira P., Cabral J., Seruca R., Oliveira C., Morgado-Díaz J.A., Reis C.A., Pinho S.S. (2013). Insulin/IGF-I Signaling Pathways Enhances Tumor Cell Invasion through Bisecting GlcNAc N-glycans Modulation. An Interplay with E-Cadherin. PLoS ONE.

[66] Carvalho S., A Catarino T., Dias A.M., Kato M., Almeida A., Hessling B., Figueiredo J., Gärtner F., Sanches J.M., Ruppert T. (2016). Preventing E-cadherin aberrant N-glycosylation at Asn-554 improves its critical function in gastric cancer. Oncogene.

[67] Chen W., Gao D., Xie L., Wang A., Zhao H., Guo C., Sun Y., Nie Y., Hong A., Xiong S. (2020). SCF-FBXO24 regulates cell proliferation by mediating ubiquitination and degradation of PRMT6. Biochem. Biophys. Res. Commun..

[68] Kari V., Raul S.K., Henck J.M., Kitz J., Kramer F., Kosinsky R.L., Übelmesser N., Mansour W.Y., Eggert J., Spitzner M. (2019). The histone methyltransferase DOT1L is required for proper DNA damage response, DNA repair, and modulates chemotherapy responsiveness. Clin. Epigenetics.

[69] Van der Post R.S., Vogelaar I.P., Carneiro F., Guilford P., Huntsman D., Hoogerbrugge N., Caldas C., Schreiber K.E.C., Hardwick R.H., Ausems M.G.E.M. (2015). Hereditary diffuse gastric cancer: Updated clinical guidelines with an emphasis on germline *CDH1* mutation carriers. J. Med. Genet..

[70] Gullo I., Devezas V., Baptista M., Garrido L., Castedo S., Morais R., Wen X., Rios E., Pinheiro J., Pinto-Ribeiro I. (2018). Phenotypic heterogeneity of hereditary diffuse gastric cancer: Report of a family with early-onset disease. Gastrointest. Endosc..

[71] Stoll J., Kupfer S.S. (2019). Risk Assessment and Genetic Testing for Inherited Gastrointestinal Syndromes. Gastroenterol. Hepatol..

[72] Brooks-Wilson A.R., Kaurah P., Suriano G., Leach S., Senz J., Grehan N., Butterfield Y.S.N., Jeyes J., Schinas J., Bacani J. (2004). Germline E-cadherin mutations in hereditary diffuse gastric cancer: Assessment of 42 new families and review of genetic screening criteria. J. Med. Genet..

[73] Guilford P., Humar B., Blair V. (2010). Hereditary diffuse gastric cancer: Translation of CDH1 germline mutations into clinical practice. Gastric Cancer.

[74] Oliveira C. The CDH1 Gene Homepage. https://databases.lovd.nl/shared/genes/CDH1.

[75] Lee K., Krempely K., Roberts M.E., Anderson M.J., Carneiro F., Chao E., Dixon K., Figueiredo J., Ghosh R., Huntsman D. (2018). Specifications of the ACMG/AMP variant curation guidelines for the analysis of germline *CDH1* sequence variants. Hum. Mutat..

[76] Rivera-Muñoz E.A., Milko L.V., Harrison S.M., Azzariti D.R., Kurtz C.L., Lee K., Mester J.L., Weaver M.A., Currey E., Craigen W. (2018). ClinGen Variant Curation Expert Panel experiences and standardized processes for disease and gene-level specification of the ACMG/AMP guidelines for sequence variant interpretation. Hum. Mutat..

[77] Hakkaart C., Ellison-Loschmann L., Day R., Sporle A., Koea J., Harawira P., Cheng S., Gray M., Whaanga T., Pearce N. (2019). Germline CDH1 mutations are a significant contributor to the high frequency of early-onset diffuse gastric cancer cases in New Zealand Māori. Fam. Cancer.

[78] Obermair F., Rammer M., Burghofer J., Malli T., Schossig A., Wimmer K., Kranewitter W., Mayrbaeurl B., Duba H.-C., Webersinke G. (2019). Cleft lip/palate and hereditary diffuse gastric cancer: Report of a family harboring a CDH1 c.687 + 1G > A germline mutation and review of the literature. Fam. Cancer.

[79] Pilonis N.D., Tischkowitz M., Fitzgerald R.C., di Pietro M. (2021). Hereditary Diffuse Gastric Cancer: Approaches to Screening, Surveillance, and Treatment. Annu. Rev. Med..

[80] Spoto C.P., Gullo I., Carneiro F., Montgomery E.A., Brosens L.A. (2018). Hereditary gastrointestinal carcinomas and their precursors: An algorithm for genetic testing. Semin. Diagn. Pathol..

[81] Kaurah P., Huntsman D., Adam M.P., Mirzaa G.M., Pagon R.A., Wallace S.E., Bean L.J.H., Gripp K.W., Amemiya A. (2002). Hereditary Diffuse Gastric Cancer. GeneReviews^®^.

[82] Fecteau H., Vogel K.J., Hanson K., Morrill-Cornelius S. (2014). The Evolution of Cancer Risk Assessment in the Era of Next Generation Sequencing. J. Genet. Couns..

[83] Robson M. (2014). Multigene Panel Testing: Planning the Next Generation of Research Studies in Clinical Cancer Genetics. J. Clin. Oncol..

[84] Lowstuter K., Espenschied C.R., Sturgeon D., Ricker C., Karam R., LaDuca H., Culver J.O., Dolinsky J.S., Chao E., Sturgeon J. (2017). Unexpected *CDH1* Mutations Identified on Multigene Panels Pose Clinical Management Challenges. JCO Precis. Oncol..

[85] Katona B.W., Clark D.F., Domchek S.M. (2020). CDH1 on Multigene Panel Testing: Look Before You Leap. J. Natl. Cancer Inst..

[86] Richards S., Aziz N., Bale S., Bick D., Das S., Gastier-Foster J., Grody W.W., Hegde M., Lyon E., Spector E. (2015). Standards and guidelines for the interpretation of sequence variants: A joint consensus recommendation of the American College of Medical Genetics and Genomics and the Association for Molecular Pathology. Genet. Med..

[87] Li M.M., Datto M., Duncavage E.J., Kulkarni S., Lindeman N.I., Roy S., Tsimberidou A.M., Vnencak-Jones C.L., Wolff D.J., Younes A. (2017). Standards and Guidelines for the Interpretation and Reporting of Sequence Variants in Cancer: A Joint Consensus Recommendation of the Association for Molecular Pathology, American Society of Clinical Oncology, and College of American Pathologists. J. Mol. Diagn..

[88] Masciari S., Larsson N., Senz J., Boyd N., Kaurah P., Kandel M.J., Harris L.N., Pinheiro H.C., Troussard A., Miron P. (2007). Germline E-cadherin mutations in familial lobular breast cancer. J. Med. Genet..

[89] Ciriello G., Gatza M.L., Beck A.H., Wilkerson M.D., Rhie S.K., Pastore A., Zhang H., McLellan M., Yau C., Kandoth C. (2015). Comprehensive Molecular Portraits of Invasive Lobular Breast Cancer. Cell.

[90] Syngal S., E Brand R., Church J.M., Giardiello F.M., Hampel H.L., Burt R.W. (2015). ACG Clinical Guideline: Genetic Testing and Management of Hereditary Gastrointestinal Cancer Syndromes. Am. J. Gastroenterol..

[91] Guidelines® N.C.P.G.i.O.N. Genetic/Familial High-Risk Assessment: Breast, Ovarian, and Pancreatic. https://www.nccn.org/professionals/physician_gls/pdf/genetics_bop.pdf.

[92] Benusiglio P.R., Malka D., Rouleau E., De Pauw A., Buecher B., Noguès C., Fourme E., Colas C., Coulet F., Warcoin M. (2013). *CDH1* germline mutations and the hereditary diffuse gastric and lobular breast cancer syndrome: A multicentre study. J. Med. Genet..

[93] Petridis C., Shinomiya I., Kohut K., Gorman P., Caneppele M., Shah V., Troy M., E Pinder S., Hanby A., Tomlinson I. (2014). Germline CDH1 mutations in bilateral lobular carcinoma in situ. Br. J. Cancer.

[94] Frebourg T., Oliveira C., Hochain P., Karam R., Manouvrier S., Graziadio C., Vekemans M., Hartmann A., Baert-Desurmont S., Alexandre C. (2006). Cleft lip/palate and CDH1/E-cadherin mutations in families with hereditary diffuse gastric cancer. J. Med. Genet..

[95] Kluijt I., Siemerink E.J., Ausems M.G., van Os T.A., de Jong D., Simões-Correia J., van Krieken J.H., Ligtenberg M.J., Figueiredo J., van Riel E. (2012). CDH1-related hereditary diffuse gastric cancer syndrome: Clinical variations and implications for counseling. Int. J. Cancer.

[96] Pattison S., Mitchell C., Lade S., Leong T., Busuttil R.A., Boussioutas A. (2017). Early relapses after adjuvant chemotherapy suggests primary chemoresistance in diffuse gastric cancer. PLoS ONE.

[97] Wagner A.D., Syn N.L., Moehler M., Grothe W., Yong W.P., Tai B.-C., Ho J., Unverzagt S. (2017). Chemotherapy for advanced gastric cancer. Cochrane Database Syst. Rev..

[98] Kang Y.-K., Shin D.-B., Chen J., Xiong J., Wang J., Lichinitser M., Guan Z., Khasanov R., Zheng L., Philco-Salas M. (2009). Capecitabine/cisplatin versus 5-fluorouracil/cisplatin as first-line therapy in patients with advanced gastric cancer: A randomised phase III noninferiority trial. Ann. Oncol..

[99] El Rami F.E., Barsoumian H.B., Khneizer G.W. (2020). Hereditary diffuse gastric cancer therapeutic roadmap: Current and novel approaches in a nutshell. Ther. Adv. Med. Oncol..

[100] Koea J.B., Karpeh M.S., Brennan M.F. (2000). Gastric Cancer in Young Patients: Demographic, Clinicopathological, and Prognostic Factors in 92 Patients. Ann. Surg. Oncol..

[101] Stiekema J., Cats A., Kuijpers A., van Coevorden F., Boot H., Jansen E., Verheij M., Ponz O.B., Hauptmann M., van Sandick J. (2013). Surgical treatment results of intestinal and diffuse type gastric cancer. Implications for a differentiated therapeutic approach?. Eur. J. Surg. Oncol..

[102] Chen W., Zheng R., Baade P.D., Zhang S., Zeng H., Bray F., Jemal A., Yu X.Q., He J. (2016). Cancer statistics in China, 2015. CA Cancer J. Clin..

[103] Howlader N., Krapcho M., Miller D. (1975–2014). SEER Cancer Statistics Review. https://seer.cancer.gov/csr/1975_2014/.

[104] Liedman B., Andersson H., Bosaeus I., Hugosson I., Lundell L. (1997). Changes in body composition after gastrectomy: Results of a controlled, prospective clinical trial. World J. Surg..

[105] Schauer M., Peiper M., Theisen J., Knoefel W. (2011). Prognostic factors in patients with diffuse type gastric cancer (linitis plastica) after operative treatment. Eur. J. Med. Res..

[106] Bang Y.-J., Van Cutsem E., Feyereislova A., Chung H.C., Shen L., Sawaki A., Lordick F., Ohtsu A., Omuro Y., Satoh T. (2010). Trastuzumab in combination with chemotherapy versus chemotherapy alone for treatment of HER2-positive advanced gastric or gastro-oesophageal junction cancer (ToGA): A phase 3, open-label, randomised controlled trial. Lancet.

[107] Al-Batran S.-E., Hartmann J.T., Probst S., Schmalenberg H., Hollerbach S., Hofheinz R., Rethwisch V., Seipelt G., Homann N., Wilhelm G. (2008). Phase III Trial in Metastatic Gastroesophageal Adenocarcinoma with Fluorouracil, Leucovorin Plus Either Oxaliplatin or Cisplatin: A Study of the Arbeitsgemeinschaft Internistische Onkologie. J. Clin. Oncol..

[108] Herzberg B., Campo M.J., Gainor J.F. (2017). Immune Checkpoint Inhibitors in Non-Small Cell Lung Cancer. Oncol..

[109] Kole C., Charalampakis N., Tsakatikas S., Vailas M., Moris D., Gkotsis E., Kykalos S., Karamouzis M.V., Schizas D. (2020). Immunotherapy for Hepatocellular Carcinoma: A 2021 Update. Cancers.

[110] Schizas D., Charalampakis N., Kole C., Economopoulou P., Koustas E., Gkotsis E., Ziogas D., Psyrri A., Karamouzis M.V. (2020). Immunotherapy for pancreatic cancer: A 2020 update. Cancer Treat. Rev..

[111] Schizas D., Charalampakis N., Kole C., Mylonas K.S., Katsaros I., Zhao M., A Ajani J., Psyrri A., Karamouzis M.V., Liakakos T. (2020). Immunotherapy for esophageal cancer: A 2019 update. Immunotherapy.

[112] Koury J., Lucero M., Cato C., Chang L., Geiger J., Henry D., Hernandez J., Hung F., Kaur P., Teskey G. (2018). Immunotherapies: Exploiting the Immune System for Cancer Treatment. J. Immunol. Res..

[113] Pardoll D.M. (2012). The blockade of immune checkpoints in cancer immunotherapy. Nat. Rev. Cancer.

[114] Janjigian Y.Y., Shitara K., Moehler M., Garrido M., Salman P., Shen L., Wyrwicz L., Yamaguchi K., Skoczylas T., Campos Bragagnoli A. (2021). First-line nivolumab plus chemotherapy versus chemotherapy alone for advanced gastric, gastro-oesophageal junction, and oesophageal adenocarcinoma (CheckMate 649): A randomised, open-label, phase 3 trial. Lancet.

[115] Le D.T., Durham J.N., Smith K.N., Wang H., Bartlett B.R., Aulakh L.K., Lu S., Kemberling H., Wilt C., Luber B.S. (2017). Mismatch repair deficiency predicts response of solid tumors to PD-1 blockade. Science.

[116] Chen I., Mathews-Greiner L., Li D., Abisoye-Ogunniyan A., Ray S., Bian Y., Shukla V., Zhang X., Guha R., Thomas C. (2017). Transcriptomic profiling and quantitative high-throughput (qHTS) drug screening of CDH1 deficient hereditary diffuse gastric cancer (HDGC) cells identify treatment leads for familial gastric cancer. J. Transl. Med..

[117] Beetham H., Chen A., Telford B.J., Single A., Jarman K.E., Lackovic K., Luxenburger A., Guilford P. (2019). A high-throughput screen to identify novel synthetic lethal compounds for the treatment of E-cadherin-deficient cells. Sci. Rep..

[118] Yang L., Wang Y., Wang H. (2019). Use of immunotherapy in the treatment of gastric cancer. Oncol. Lett..

[119] Sun H., He S., Meng L., Wang Y., Zhang H., Liu Y., Wang J., Tao M., Barta S.K., Dulaimi E. (2019). Engineering of Chimeric Antigen Receptor T Cells with integrin αEβ7 Results in Augmented Therapeutic Efficacy against E-cadherin positive tumor. BioRxiv.

[120] Chou Y.-Y., Jeng Y.-M., Lee T.-T., Hu F.-C., Kao H.-L., Lin W.-C., Lai P.-L., Hu R.-H., Yuan R.-H. (2007). Cytoplasmic CD24 Expression Is a Novel Prognostic Factor in Diffuse-Type Gastric Adenocarcinoma. Ann. Surg. Oncol..

[121] Panagiotou E., Syrigos N.K., Charpidou A., Kotteas E., Vathiotis I.A. (2022). CD24: A Novel Target for Cancer Immunotherapy. J. Pers. Med..

[122] Yang L., Li A., Lei Q., Zhang Y. (2019). Tumor-intrinsic signaling pathways: Key roles in the regulation of the immunosuppressive tumor microenvironment. J. Hematol. Oncol..

[123] Liu Y., Di S., Shi B., Zhang H., Wang Y., Wu X., Luo H., Wang H., Li Z., Jiang H. (2019). Armored Inducible Expression of IL-12 Enhances Antitumor Activity of Glypican-3–Targeted Chimeric Antigen Receptor–Engineered T Cells in Hepatocellular Carcinoma. J. Immunol..

[124] Chen J., Xie Z.-R., Wu Y. (2014). Computational Modeling of the Interplay between Cadherin-Mediated Cell Adhesion and Wnt Signaling Pathway. PLoS ONE.

[125] Yang D., Zhao D., Chen X. (2017). MiR-133b inhibits proliferation and invasion of gastric cancer cells by up-regulating FBN1 expression. Cancer Biomark..

[126] Li J., Zhang H., Bei S., Zhang X., Li H., Ye L., Feng L. (2022). Disruption of Wnt/β-catenin Pathway Elevates the Sensitivity of Gastric Cancer Cells to PD-1 Antibody. Curr. Mol. Pharmacol..

